# The effect of layer thickness ratio on the drug release behavior of alternating layered composite prepared by layer-multiplying co-extrusion

**DOI:** 10.3389/fbioe.2023.1217938

**Published:** 2023-06-23

**Authors:** Huiyu Zheng, Cong Zhang, Guiting Liu, Rong Chen, Shaoyun Guo

**Affiliations:** The State Key Laboratory of Polymer Materials Engineering, Polymer Research Institute of Sichuan University, Chengdu, China

**Keywords:** layered structures, interface, extrusion, drug release, scaffold

## Abstract

Multi-layered drug delivery (MLDD) system has promising potential to achieve controlled release. However, existing technologies face difficulties in regulating the number of layers and layer-thickness ratio. In our previous works, layer-multiplying co-extrusion (LMCE) technology was applied to regulate the number of layers. Herein, we utilized layer-multiplying co-extrusion technology to modulate the layer-thickness ratio to expand the application of LMCE technology. Four-layered poly (ε-caprolactone)-metoprolol tartrate/poly (ε-caprolactone)-polyethylene oxide (PCL-MPT/PEO) composites were continuously prepared by LMCE technology, and the layer-thickness ratios for PCL-PEO layer and PCL-MPT layer were set to be 1:1, 2:1, and 3:1 just by controlling the screw conveying speed. The *in vitro* release test indicated that the rate of MPT release increased with decreasing the thickness of the PCL-MPT layer. Additionally, when PCL-MPT/PEO composite was sealed by epoxy resin to eliminate the edge effect, sustained release of MPT was achieved. The compression test confirmed the potential of PCL-MPT/PEO composites as bone scaffolds.

## 1 Introduction

Controlled release is the ultimate goal of drug delivery. Many strategies have been developed to fabricate drug delivery systems with desirable drug release behaviors, such as stimulus-responsive drug delivery systems (Antoniou et al., 2021), nano drug delivery systems (Birk et al., 2021) and multi-layered drug delivery systems (Abdul and Poddar, 2004). Among these, multi-layered drug delivery (MLDD) systems are garnering increasing attention due to their inherent flexibility and controllability in drug release, extensive applicability, and the potential for continuous preparation (Abdul and Poddar, 2004; Choi and Jeong, 2011; Choi et al., 2013). Nowadays, MLDD systems can be prepared by layer-by-layer (LBL) self-assembly technology (Alkekhia et al., 2020), microfluidic technology (Liu et al., 2018), electrospinning technology (Laha et al., 2017) and lamination (Mazel and Tchoreloff, 2021). Moreover, their release behaviors can be adjusted flexibly and controllably by manipulating the number of layers, layer thickness ratio and drug distribution (Heng, 2018; Cerea et al., 2020; Finsgar et al., 2021). For example, Rebeca Hernández et al. utilized LBL technology to construct alginate/chitosan multi-layered complex systems, in which tamoxifen was loaded in an intermediate position. The release of tamoxifen was regulated by varying the number of alternating layers (Criado-Gonzalez et al., 2019). In another study, Michael Kai Tsun To et al. prepared poly (lactic-co-glycolic acid)/alginate core-shell double-layer rifampicin delivery system by microfluidic technology (Wu et al., 2013). The release kinetics of rifampicin were adjusted by manipulating the thickness ratio of the core-shell structure, resulting in the achievement of a near-zero-order release pattern.

The design strategy of multi-layered structures has provided a versatile platform for the fabrication of drug delivery systems with programmed release behaviors. However, it is difficult for the existing preparation technology to realize the continuous preparation of multi-layered drug delivery systems with high layer numbers and wide layer thickness ratio at the same time. For instance, LBL technology can produce a high-layer number drug delivery system, but it encounters difficulties in achieving flexible regulation of layer thickness ratio and ensuring continuous preparation. On the other hand, the microfluidic technology can achieve flexible control of layer thickness ratio and continuous preparation of drug delivery systems. Nevertheless, it faces challenges in constructing layered structures with a high number of layers. Electrostatic spinning technology and laminating technology face difficulties in building high-leveler number of drug delivery systems. Our previous works developed layer-multiplying co-extrusion (LMCE) technology to continuously construct composites with thousands of alternating multi-layers (Ji et al., 2019; Zhang et al., 2019; Zheng et al., 2020). By this technology, the two components are each heat-processed and conveyed through a screw extruder and stacked as a two-layer product via a co-extrusion block. The number of layers of the product can be doubled with an application of layer multiplying elements (LMEs), and the layer thickness ratio of the two components can be adjusted by the ratio of screw extruder delivery rate. The LMCE technology has showed the potential of preparing multi-layered drug delivery systems with high layer number and wide layer thickness ratio. Motivated by this, we utilized LMCE technology to continuously construct alternating multi-layered drug delivery systems with a layer-thickness ratio of 1:1, including 128 layers, and investigated the effect of the number of layers on the drug release behavior (Liu et al., 2022; Zhang et al., 2022).

**FIGURE 1:**
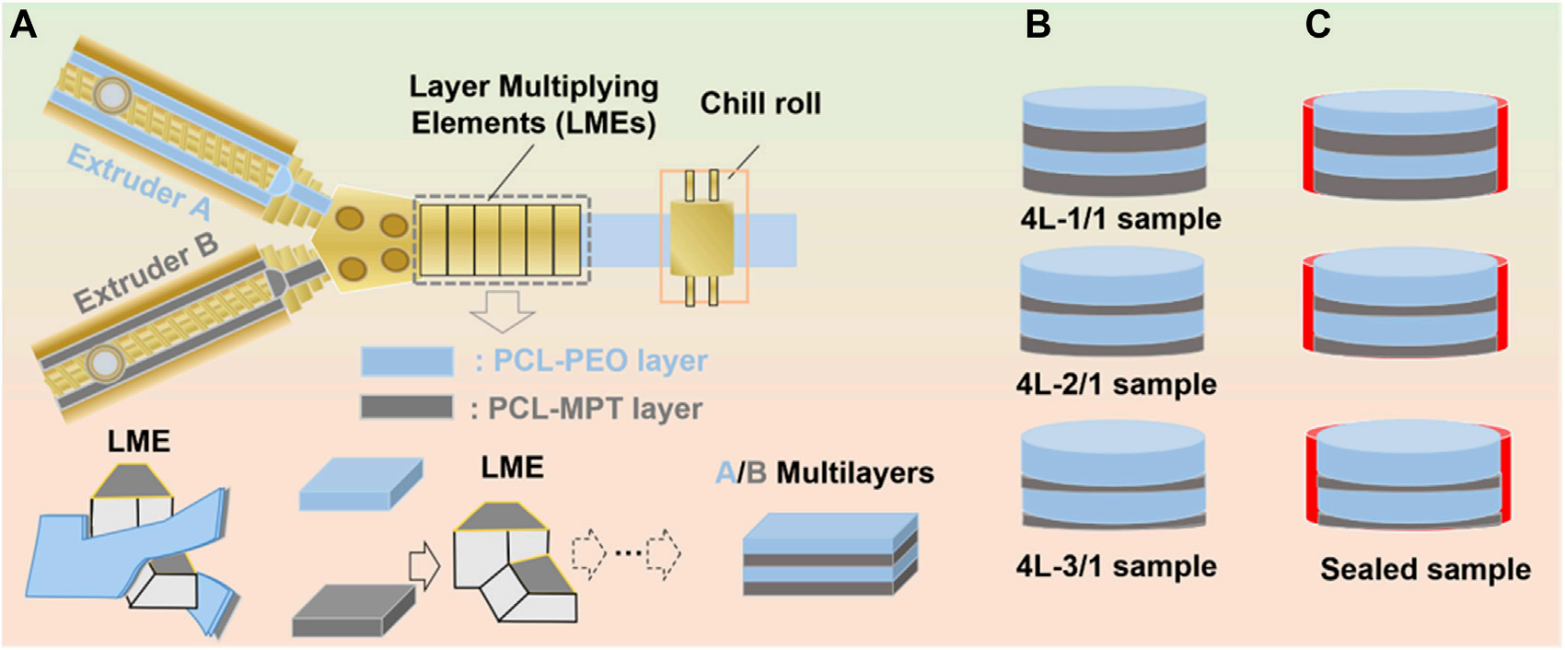
Schematic of layer-multiplying co-extrusion system **(A)**, prepared four-layered PCL-MPT/PEO composites with different layer-thickness ratio **(B)** and sealed PCL-MPT/PEO composites **(C)**.

In this work, we further investigated the effect of layer thickness ratio on drug release behavior to expand the application of LMCE technology in the preparation of multi-layered drug delivery systems. Poly (ε-caprolactone) (PCL) worked as the loading layer of metoprolol tartrate (MPT), and PCL-polyethylene oxide (PEO) composite layer acted as the barrier layer. Three four-layered PCL-MPT/PEO composites with layer thickness ratios of 1:1, 2:1 and 3:1 for PCL-PEO layer and PCL-MPT layer, respectively, were prepared by LMCE technology (Figure 1A). *In vitro* release tests were carried out and the release mechanism was analyzed by the combination of scanning electron microscope and Raman spectroscopy. In addition, compression performance tests were performed to explore the potential of the PCL-MPT/PEO composites as tissue engineering scaffolds.

## 2 Materials and method

### 2.1 Materials

Poly (ε-caprolactone) (PCL, Mw = 80,000) was provided by Perstorp Co. Polyethylene oxide (PEO, Mw = 100,000) was purchased from Sumitomo Chemical Co. Metoprolol tartrate (MPT) and phosphate buffered solution (PBS) were supplied by Guangzhou Hanfang Pharmaceutical Co. and Zhongshan Golden Bridge Biotechnology Co., respectively.

### 2.2 Preparation of four-layered PCL-MPT/PEO composites by LMCE technology

PCL, PEO and MPT were dried in a vacuum oven at 40°C for 24 h, then mixed in a high mixer according to the mass ratio shown in Table 1. Subsequently, components were added to a twin-screw extruder (screw diameter: 20 mm, length diameter (L/D) ratio: 40) for melt extrusion and pelletizing. The two sections of extrusion screw and die temperatures were set at 40°C, 90°C, and 130°C, respectively, and the screw speed was 130 rpm. After pelletizing, the product was dried under vacuum for further application.

**TABLE 1:** Weight ratio of PEO, PCL and MPT in PCL-MPT and PCL-PEO samples.

Abbreviation	PCL (w_t_%)	PEO (w_t_%)	MPT (w_t_%)
PCL-MOT	90	0	10
PCL-PEO	50	50	0

The dried PCL-MPT sample and PCL-PEO sample were added to two single-screw extruders of the LMCE equipment. The temperatures of screw section were set at 40°C, 90°C, and 130°C from inlet to outlet, respectively. The temperatures of co-extrusion block and layer multiplying elements (LMEs) were set at 130°C. The four-layered PCL-MPT/PEO composites were prepared by the connection between co-extrusion block and LMEs. The layer thickness ratio of PCL-MPT layer and PCL-PEO layer was adjusted by the screw speed ratio. In this work, three four-layered PCL-MPT/PEO composites with layer thickness ratios of 1:1, 2:1, and 3:1 for PCL-PEO layer and PCL-MPT layer, respectively, were prepared. A 10 mm diameter cutter was used to cut the PCL-MPT/PEO composites into small rounds with a diameter of 10 mm and a thickness of 2.0 mm (Figure 1B). The samples were named as shown in Table 2. Taking “4L-2/1” as an example, “4 L” indicated that the number of layers of samples was 4, “2/1” referred to the thickness ratio of PCL/PEO layer and PCL-MPT layer was 2:1.

**TABLE 2:** The abbreviation of PCL-MPT/PEO composites.

Abbreviation	Layer number	Thickness ratio of PCL-PEO layer and PCL-MPT layer
4L-1/1	4	1:1
4L-2/1	4	2:1
4L-3/1	4	3:1

### 2.3 The preparation of sealed samples

The surface PCL-PEO layer of pre-cut 4L-1/1, 4L-2/1 and 4L-3/1 samples was sealed with tape. Then samples were placed flat in a 20 mm diameter round plastic mold with the top PCL-MPT layer and the bottom PCL-PEO layer sealed with tape. Pre-configured epoxy resin was added around the samples drop by drop until the epoxy resin was level with the upper surface of the samples. The plastic mold was placed in a vacuum oven at 40°C for 7 days. Then cured samples were removed from the mold and the tape at the PCL-PEO layer was removed. Thus, sealed PCL-MPT/PEO composites were obtained (Figure 1C). The samples were named as 4L-1/1-1, 4L-2/1-1, and 4L-3/1-1.

### 2.4 Morphology observation

The multi-layered structures of samples were observed by a polarizing microscope (POM) (Olympus BX51). The morphology of 4L-1/1 sample soaked in PBS solution for 2 weeks was observed by scanning electron microscope (XL30FZG, Philips) with an accelerating voltage of 20 kV. Before testing, the samples were dried to constant weight in a vacuum oven at 40°C, then quenched in liquid nitrogen. The sections were vacuum gilded.

### 2.5 *In vitro* release test

The sample slices were immersed in 20 mL of PBS buffer and stirred at 100 rpm in an incubator at 37°C. The PBS buffer was removed completely at regular intervals and 20 mL of new PBS buffer was added. The dissolved concentration of MPT in PBS buffer was tested by UV-1750 UV-Vis spectrophotometer (Shimadzu, Japan) at 222 nm, and the applicable concentration range of the standard curve equation for MPT was 0.5–15.0 μg/mL, and the drug concentration and absorbance showed a good linear relationship (R > 0.999) in the applicable range. The experiment was repeated three times for all samples.

### 2.6 Raman spectroscopy

The 4L-1/1-1 samples were soaked in PBS buffer for 2, 4, and 8 h, respectively, then removed and dried with filter paper, quenched with liquid nitrogen. The cross-section of sample was observed by a DXRxi microlaser Raman spectrometer (Thermo Fisher, United States) equipped with an Olympus BX51 optical microscope (wavelength of 780 nm, resolution of 0.4 cm^–1^, step size of 3 μm, 10 scans and exposure time of 2 Hz). The Raman curve of MPT was used as the standard curve, and the Correlation mode was selected to image different areas of the sample (area 50 μm × 50 μm).

### 2.7 The compression performance test

Samples were soaked in PBS buffer for 2 weeks, and then removed and dried with filter paper. Five samples were stacked and tested on a universal material testing machine (CM-4104, MTS Systems Co., United States) with a compression speed of 1 mm/min and a maximum compression strain of 60%. The test environment was 23°C and 55% humidity. Three samples were tested for each group and the results were averaged.

## 3 Results and discussion

### 3.1 Morphology observation

**FIGURE 2:**
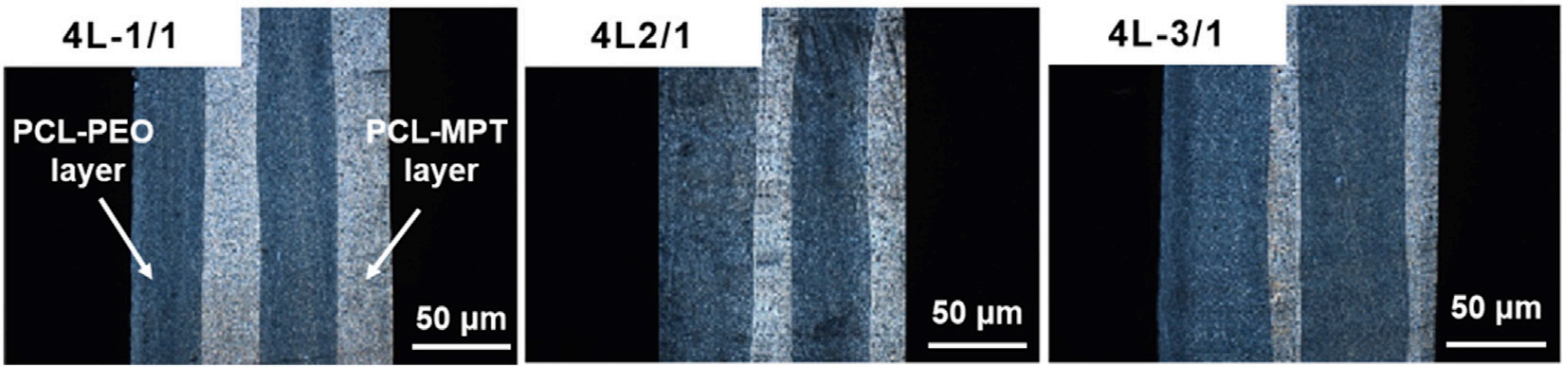
POM micrographs of PCL-MPT/PEO composites with different layer thickness ratios.

In this work, PCL-MPT/PEO composites with four-layered structure were continuously prepared by LMCE technology, the mechanism of which was described in detail in our previous work (Liu et al., 2022; Zhang et al., 2022). The two-layered PCL-MPT/PEO composites were prepared by the assembly of co-extrusion block, then four-layered PCL-MPT/PEO composites were obtained by an application of LMEs. The layer thickness ratio of PCL-MPT layer and PCL-PEO layer was adjusted by the screw speed ratio (Figure 1). The morphology of PCL-MPT/PEO composites was observed by POM and presented in Figure 2. The darker layer was the PCL-PEO layer and the brighter layer was the PCL-MPT layer. The four-layered PCL-MPT/PEO composites with layer thickness ratios of 1:1, 2:1, and 3:1 for PCL-PEO layer and PCL-MPT layer were successfully prepared by LMCE technology. Since the thickness of PCL-MPT/PEO composite was about 2 mm, the thickness of PCL-PEO layer of 4L-1/1, 4L-2/1, and 4L-3/1 sample was about 500, 330, and 250 μm, respectively. All the samples had a good layer structure, and the PCL-PEO layer and PCL-MPT layer were arranged in a continuous alternating pattern along the extrusion direction with a regular structure.

**FIGURE 3:**
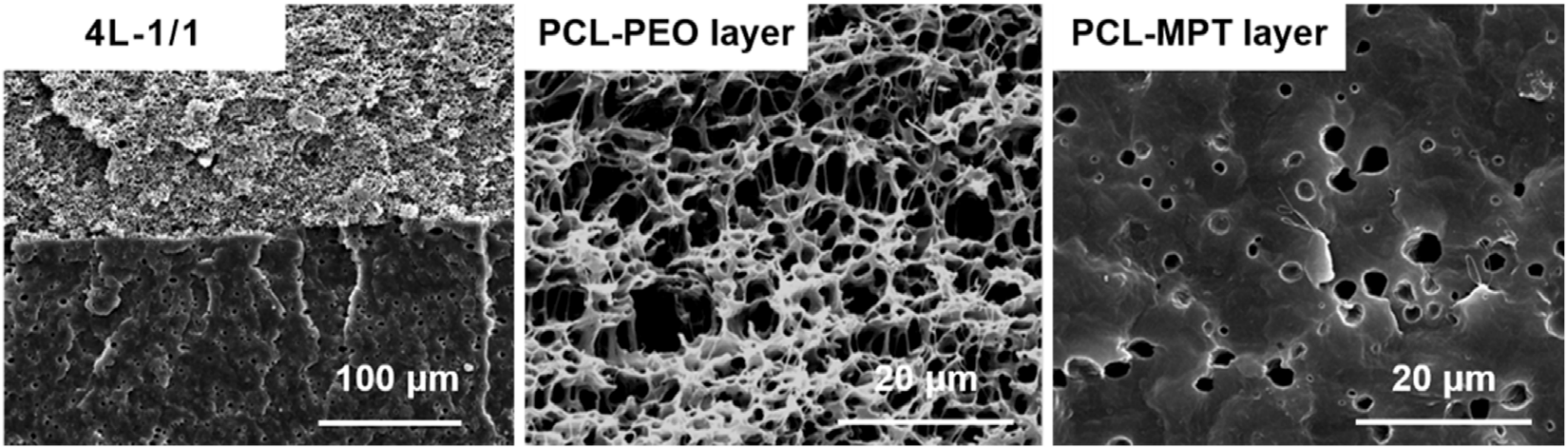
SEM images of 4L-1/1 sample after immersion for 2 weeks.

Before the evaluation of *in vitro* release, the 4L-1/1 sample was taken for SEM evaluation to observe the morphology of PCL-PEO layer and PCL-MPT layer after immersion in PBS for 2 weeks. As Figure 3 presented, interconnected pores in the PCL-PEO layer and a small number of isolated micropores (1–2 μm) in the PCL-MPT layer could be clearly observed. The pores in PCL-MPT layer were caused by the diffusion of MPT, and the pores in PCL-PEO layer were resulted by the dissolution of PEO. It could be considered that the diffusion of PEO was much faster than that of MPT.

### 3.2 *In vitro* drug release

**FIGURE 4:**
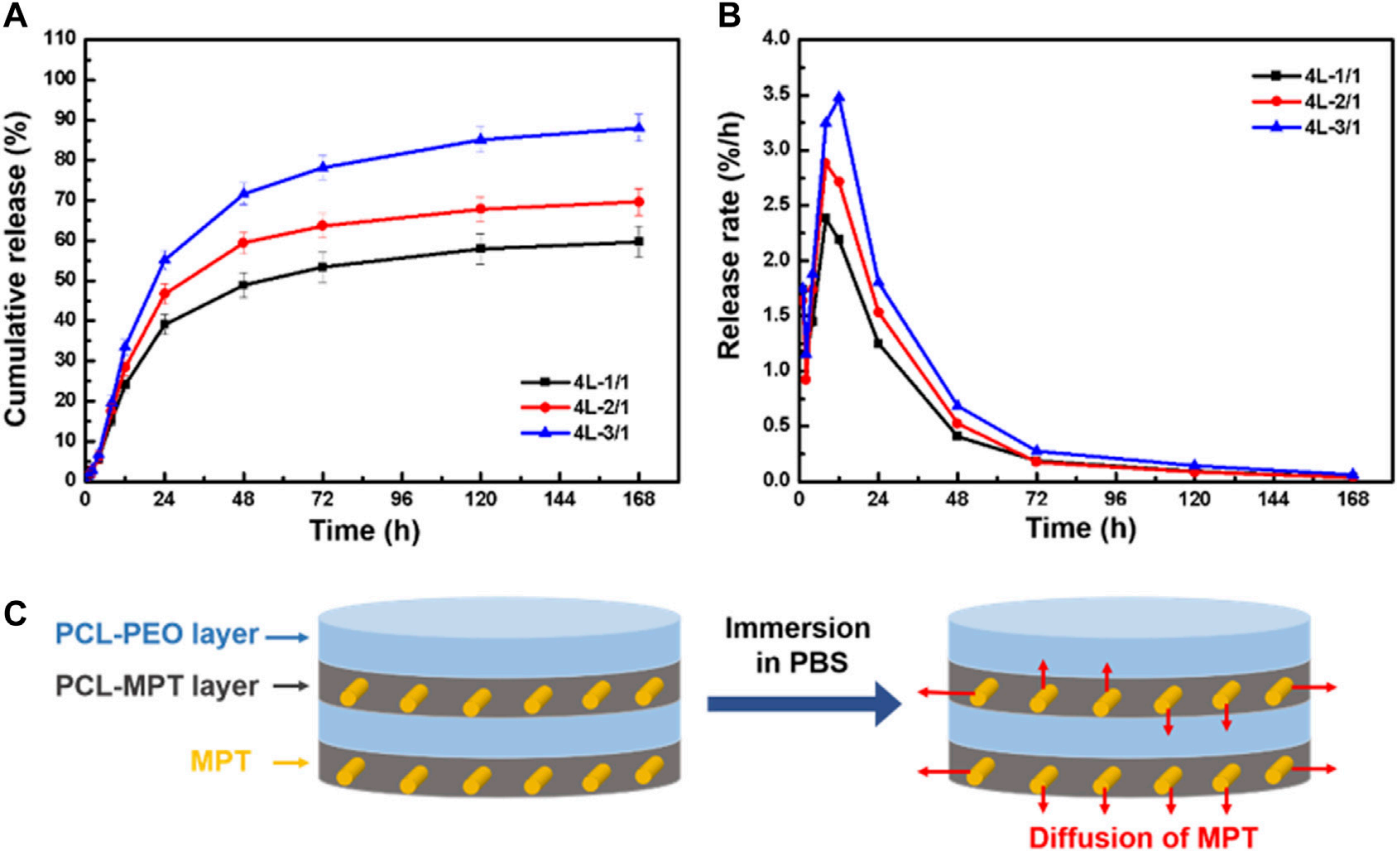
The cumulative release curve **(A)** and release rate curve **(B)** of PCL-MPT/PEO composites, and schematic of the diffusion of MPT in PCL-MPT/PEO composite after immersion in PBS **(C)**.

The cumulative release and release rate profiles of PCL-MPT/PEO composites were shown in Figure 4A and Figure 4B, respectively. In the initial 24 h, MPT release rate increased with decreasing the thickness of PCL-MPT layer. The 24-h MPT release amount of 4L-1/1, 4L-2/1, and 4L-3/1 sample was about 40%, 46%, and 55%, respectively. The theory of diffusion kinetics showed that the larger the diffusion path of the drug in the carrier, the longer the time required for complete release (Siepmann and Siepmann, 2008). In the test of *in vitro* release, PCL-MPT/PEO composites were immersed in PBS solution. The MPT of surface PCL-MPT layer was released first, and the PEO of PCL-PEO layer was dissolved at the same time, which provided the diffusion channel for the MPT of inner PCL-MPT layer (Figure 4C). As the dissolution of PEO, which could form interconnected pores in the PCL-PEO layer, was much faster than that of MPT, the rate of MPT release of PCL-MPT/PEO composite was mainly determined by the diffusion of MPT in PCL-MPT layer. This phenomenon could explain the observed increase in MPT release rate during the initial 24-h period as the thickness of the PCL-MPT layer decreases.

**FIGURE 5:**
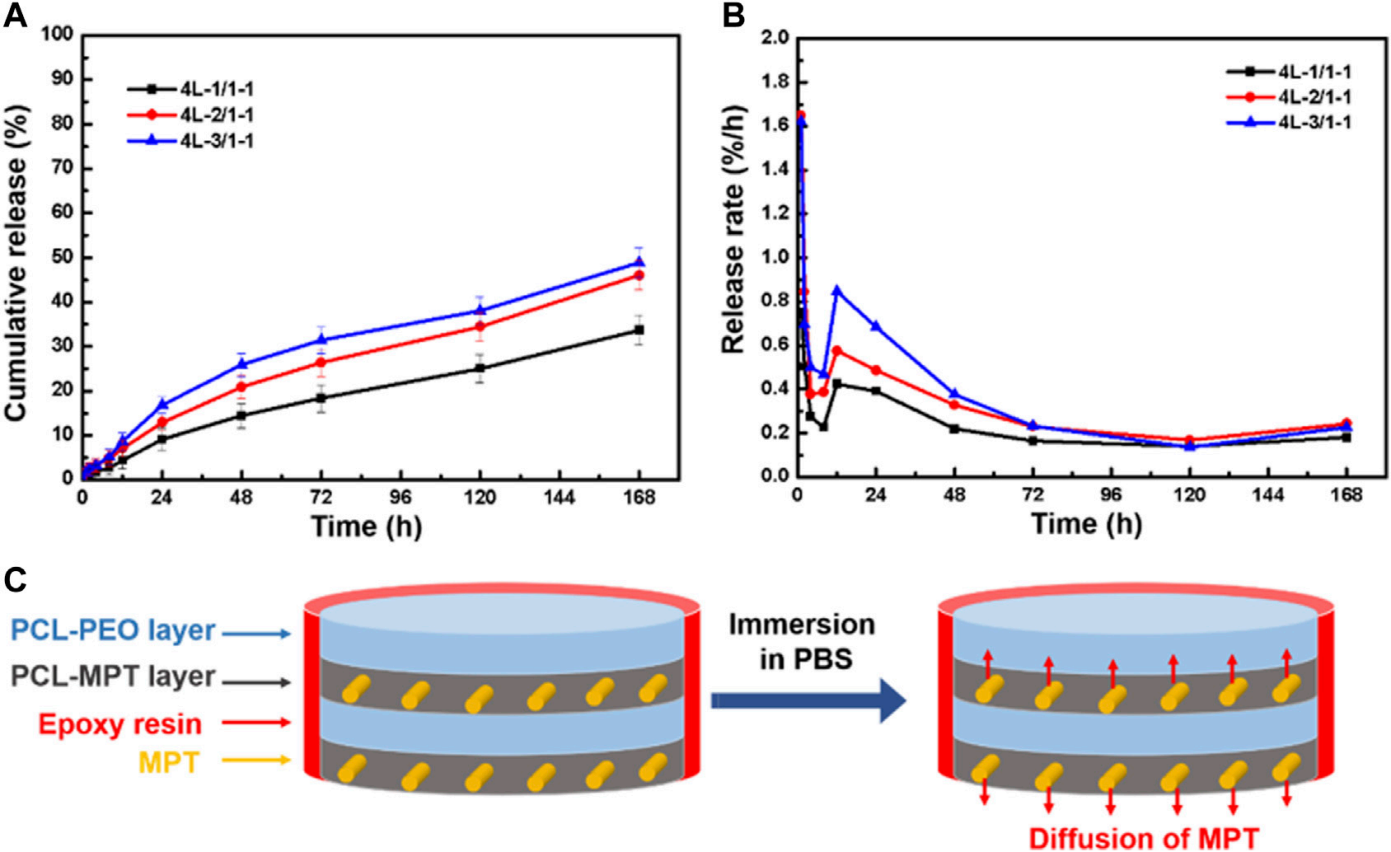
The cumulative release curve **(A)** and release rate curve **(B)** of sealed PCL-MPT/PEO composites, and schematic of the diffusion of MPT in sealed PCL-MPT/PEO composite after immersion in PBS **(C)**.

To eliminate the edge effect of sample on the MPT release, PCL-MPT/PEO composites were sealed by epoxy resin leaving the exposure of only the top and bottom surfaces. The cumulative release and release rate curves of sealed samples were presented in Figure 5A and Figure 5B, respectively. It could be seen that the MPT release rate of sealed samples were slower than that of unsealed samples. The 24-h MPT release amount of 4L-1/1-1, 4L-2/1-1, and 4L-3/1-1 sample was about 10%, 14%, and 18%, respectively. The thinner thickness of PCL-MPT layer led to the faster rate of MPT release, which was consist to MPT release behavior of unsealed samples. After the sample was sealed, MPT could only be released in the direction perpendicular to the layer plane, which prolonged the release path of MPT at the edge, resulting in a slower release rate of sealed sample compared to the unsealed sample (Figure 5C). The rate of MPT release gradually decreased from 1 to 8 h, which might be due to the diffusion of MPT in the surface PCL-MPT layer. The rate of MPT release increased from 8 to 12 h, which might be resulted by the diffusion of MPT in the inner PCL-MPT layer. After 48 h, the MPT release tended to be stable.

### 3.3 Drug release mechanism

**FIGURE 6:**
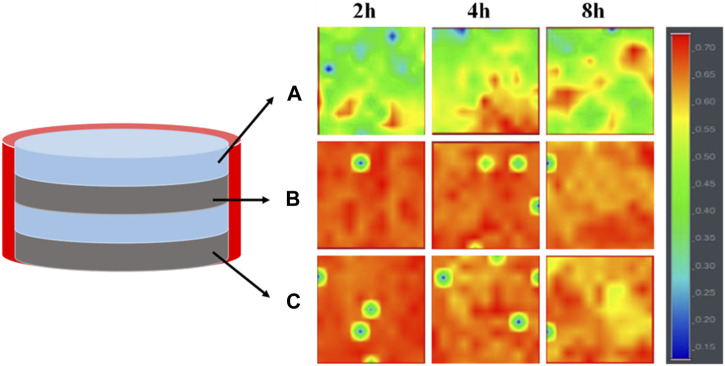
Raman spectroscopy imaging of the three different regions of 4L-1/1-1 sample immersed in PBS for different times: surface PCL-PEO layer **(A)**, inner PCL-MPT layer **(B)** and surface PCL-MPT layer **(C)**.

In order to elucidate the drug release mechanism of the sealed samples more clearly, 4L-1/1-1 samples soaked in PBS buffer for different times were subjected to Raman spectroscopy analysis (Figure 6). The results revealed that after 2 h of release, a minimal quantity of MPT from the inner PCL-MPT layer diffused into the surface PCL-PEO layer. Following 4 h of release, an increased dissolution of PEO and the formation of additional pores within the PCL-PEO layer were observed, consequently leading to a higher quantity of MPT diffusion into the surface PCL-PEO layer. At the same time, the MPT of surface PCL-MPT layer was continuously released. After 8 h of release, the MPT amount of inner PCL-MPT layer significantly decreased, which mainly diffused into the surface PCL-PEO layer. Consequently, the release of MPT occurred from both the surface PCL-MPT layer and the surface PCL-PEO layer, leading to an augmented MPT release rate during the subsequent 4-h period.

**FIGURE 7:**
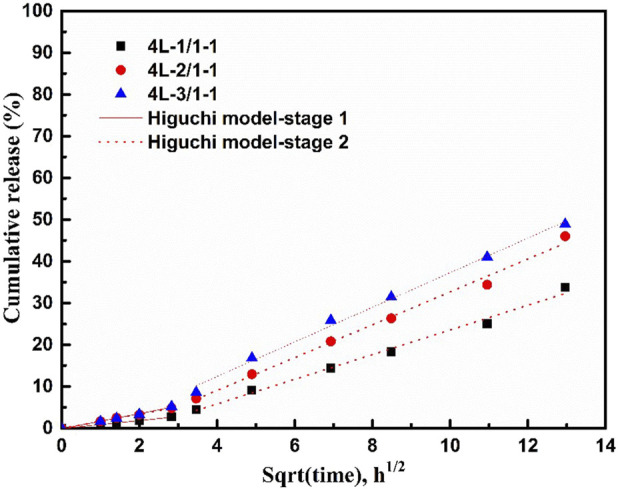
Fitting results of drug release profile of 4L-1/1-1, 4L-2/1-1, and 4L-3/1-1 samples.

Combining the release curves (Figure 5) and Raman spectroscopy analysis (Figure 6), the release process of sealed samples could be divided into two stages. The first stage was mainly the release of MPT from surface PCL-MPT layer in the initial 8 h, and the second stage was the release of MPT form both surface PCL-MPT layer and surface PCL-PEO layer, the MPT of which was diffused from the inner PCL-MPT layer. In addition, the release profiles of the two stages of sealed PCL-MPT/PEO composites were almost consistent with Higuchi release model (Figure 7; Table 3) (Lu and Hagen, 2020; Karthikeyan et al., 2021). The Higuchi constant increased with decreasing the thickness of PCL-MPT layer, which was consistent with the analysis of MPT release rate.

**TABLE 3:** Release kinetics parameters (*k*_*1*_, *k*_*2*_) and correlation coefficients, (*Rc* ^2^), for MPT released from 4L-1/1-1, 4L-2/1-1, and 4L-3/1-1 samples.

Specimen	First stage		Second stage	
	*k*_*1*_	*Rc* ^2^	*k*_*2*_	*Rc* ^2^
4L-1/1-1	0.97	0.99	2.96	0.99
4L-2/1-1	1.68	0.99	3.94	0.99
4L-3/1-1	1.82	0.99	4.15	0.99

### 3.4 Compression performance

**FIGURE 8:**
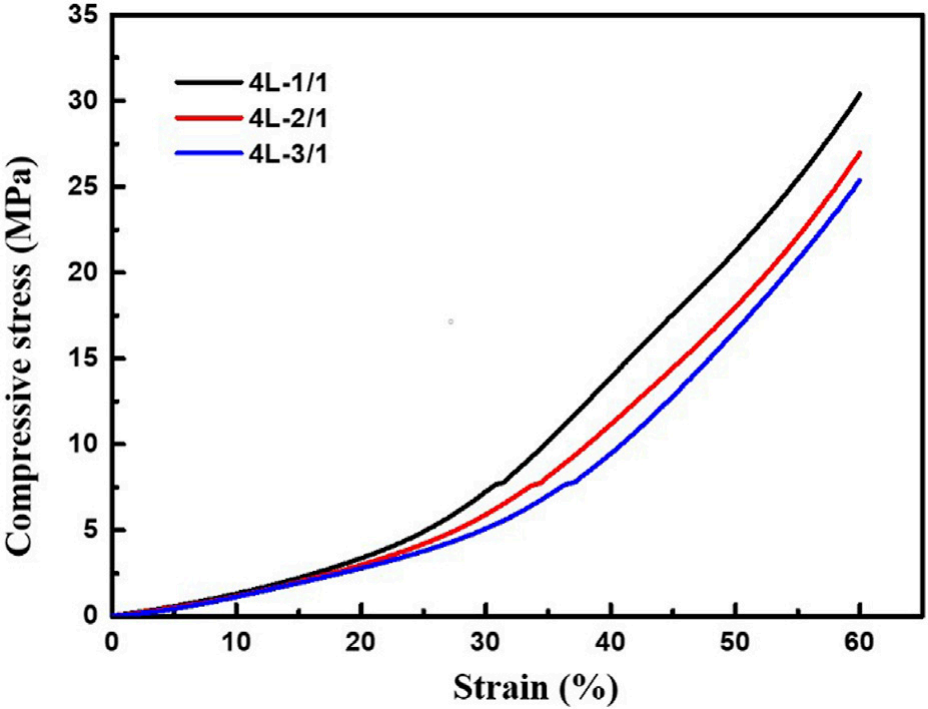
Compressive stress-strain curves of PCL-MPT/PEO composites with different layer thickness ratio.

For tissue engineering scaffolds, mechanical property evaluation, especially compression property study, was essential (Ghorbani et al., 2016). 4L-1/1, 4L-2/1, and 4L-3/1 sample after immersion in PBS for 2 weeks was taken for compression test, and the compressive stress-strain curves were presented in Figure 8. The compressive strength in 60% strain of 4L-1/1, 4L-2/1, and 4L-3/1 sample was about 30.4, 27.0, and 25.4 MPa, respectively. The compression modulus of 4L-1/1, 4L-2/1, and 4L-3/1 sample was about 15.5 MPa, 13.8 and 13.0 MPa, respectively (Table 4). The compressive strength of PCL-MPT/PEO composites decreased with increasing the thickness of PCL-PEO layer, which might be due to the fact that the forming of interconnected pores in PCL-PEO layer after immersion in PBS could disrupt the regularity of the composite structure and thus reduce the mechanical strength. The compressive performance of PCL-MPT/PEO composites suggested their potential as bone tissue scaffolds (Porter et al., 2000; Fu et al., 2013).

**TABLE 4:** Compressive properties of PCL-MPT/PEO composites.

Sample	4L-1/1	4L-2/1	4L-3/1
Compressive strength (MPa)	30.4 ± 0.8	27.0 ± 0.6	25.4 ± 0.9
Compression modulus (MPa)	15.5 ± 0.6	13.8 ± 0.5	13.0 ± 0.4

## 4 Conclusion

In this work, we continuously prepared four-layered PCL-MPT/PEO composites by LMCE technology, and the layer-thickness ratio for PCL-MPT layer and PCL-PEO layer was modulated by the screw speed ratio. To investigate the effect of layer-thickness ratio on the drug release behavior, four-layered PCL-MPT/PEO composites with thickness ratios of 1:1, 2:1 and 3:1 for PCL-PEO layer and PCL-MPT layer were prepared, respectively. *In vitro* release test results indicated that MPT release rate increased with decreasing the thickness of PCL-MPT layer in the initial 24 h, which could be contributed to the fact that the rate of MPT release of PCL-MPT/PEO composite was mainly determined by the diffusion of MPT in PCL-MPT layer. Further, PCL-MPT/PEO composites were sealed by epoxy resin to eliminate the edge effect on drug release. The MPT release rate of sealed samples were slower than that of unsealed samples, which could be resulted by the prolonged the release path of MPT at the edge of samples. Combining the release curves and Raman spectroscopy analysis, the release process of sealed samples could be divided into two stages, both of which were consistent with Higuchi release model. The sealed composites should possess the potential as controlled directional drug delivery system. In addition, the compression test confirmed the potential of four-layered composites as bone scaffold. This work should expand the application of LMCE technology in the preparation of multi-layered drug delivery systems.

## Data Availability

The original contributions presented in the study are included in the article/Supplementary Material, further inquiries can be directed to the corresponding authors.
